# Assessing the impact of Tadalafil on the sexuality and quality of life after acute urinary retention: a randomized controlled Trial

**DOI:** 10.1192/j.eurpsy.2023.2324

**Published:** 2023-07-19

**Authors:** K. Bouassida, A. Loghmari, M. Ben Othmen, G. Tlili, E. Acacha, W. Ben Abdallah, M. Kahloul, W. Hmida, R. Jaballah, A. Zorgati, R. Boufek, M. Jaidane

**Affiliations:** 1 Urology; 2 Anesthesiology department; 3 Emergency Department, Sahloul teachin Hospital Sousse, Sousse, Tunisia

## Abstract

**Introduction:**

Acute urinary retention (AUR) is known to have major impact on the quality of life and sexuality of patients with benign prostatic hyperplasia (BPH). However, little is known about specific approachs that could reduce this impact. Tadalafil, an efficient medication in uncomplicated BPH, may be also useful in serious complications, especially the AUR.

**Objectives:**

The aim of this study is to assess the impact of Tadalafil on sexuality and quality of life in patients AUR related to BPH.

**Methods:**

This is a randomized, double-blind clinical trial conducted in the Urology and Emergency Departments of Sahloul Teaching Hospital, between June 2020 and January 2022. Consenting men over the age of 45 who had an AUR complicating a BPH were included. Fifty three patients were enrolled and randomized into two groups: intervention group (n=29) in which patients received 5 mg/day of Tadalafil, and control group (n=24) in which patients received a comparable tablet of placebo. The evaluation focused on sexuality and quality of life. For theses purposes we used SF-36 short form and IIEF 15.

**Results:**

Socio-demographic characteristics were comparable between both groups. Sexuality assessed by IIEF 15 was significantly better in the intervention group (p=0.02). The four SF-36 domains were also significantly improved by Tadalafil: “Physical Function” (p=0.046), “Pain” (p=0.01), “General Health” (p=0.029) and “Health change” (p=0.02).

**Conclusions:**

Sexuality and quality of life could be improved by Tadalafil in complicated BPH.

**Disclosure of Interest:**

None Declared

